# Morning reduction of photosynthetic capacity before midday depression

**DOI:** 10.1038/srep04389

**Published:** 2014-03-17

**Authors:** Kohei Koyama, Shuhei Takemoto

**Affiliations:** 1 Department of Life Science and Agriculture, Obihiro University of Agriculture and Veterinary Medicine, Obihiro, Hokkaido 080-8555, Japan; 2 Department of Environmental Science, Ishikawa Prefectural University, Nonoichi, Ishikawa 921-8836, Japan; 3 The University of Tokyo Tanashi Forest, Graduate School of Agricultural & Life Sciences, 1-1-8 Midoricho, Nishitokyo, Tokyo 188-0002, Japan

## Abstract

Midday depression of photosynthesis has important consequences for ecosystem carbon exchange. Recent studies of forest trees have demonstrated that latent reduction of photosynthetic capacity can begin in the early morning, preceding the midday depression. We investigated whether such early morning reduction also occurs in an herbaceous species, *Oenothera biennis*. Diurnal changes of the photosynthetic light response curve (measured using a light-emitting diode) and incident sunlight intensity were measured under field conditions. The following results were obtained: (1) the light-saturated photosynthetic rate decreased beginning at sunrise; (2) the incident sunlight intensity on the leaves increased from sunrise; and (3) combining (1) and (2), the net photosynthetic rate under natural sunlight intensity increased from sunrise, reached a maximum at mid-morning, and then showed midday depression. Our results demonstrate that the latent morning reduction of photosynthetic capacity begins at sunrise, preceding the apparent midday depression, in agreement with previous studies of forest trees.

Terrestrial ecosystems in temperate regions can function as carbon sinks, thereby moderating the effects of climate change^1^,^2^. Accordingly, the response of terrestrial ecosystems to the changing climate is currently an area of major concern in the field of plant ecophysiology. Recent process-based models have successfully predicted ecosystem carbon and water exchanges and their responses to climate change^2^,^3^,^4^,^5^,^6^. For more accurate predictions of these process-based models, the dependence of carbon exchange should be evaluated for each environmental variable independently in leaf-level studies.

The daytime carbon and water exchanges of plant leaves reflect a balance between stimulation from high light exposure and depression from a high vapor pressure deficit^7^,^8^. High light exposure during the day stimulates stomata to open, thereby driving gas exchange^9^. In turn, the stomatal aperture is negatively dependent on the transpiration rate^10^, and stomata often close during the day when the humidity deficit is high^4^,^11^,^12^,^13^,^14^,^15^. Consequently, leaf gas exchange may show a midday depression on particularly sunny days^16^. This midday depression has been reported not only in dry regions^16^, but also in wet temperate regions^14^,^17^,^18^ and can reduce ecosystem-level carbon uptake^19^,^20^. Hence, understanding of midday depression at the scale of the individual leaf is necessary for improving the process-based modeling of ecosystem gas exchange. Considering the processes described above, the midday depression can be considered to result from the combination of the effects of light intensity, which positively drives photosynthesis, and other factors that reduce the photosynthetic rate by means of stomatal closure. Therefore, it is necessary to separately evaluate the effects of diurnally changing light intensity independently from other factors. One means of achieving this objective is to repeatedly measure photosynthetic light response curves independently of the external light intensity over the time course of one day. Recently, two studies of tropical rainforest tree species^21^,^22^ and one study of a temperate tree species^23^ investigated diurnal changes in photosynthetic capacity under saturating light intensity and the *in situ* photosynthetic rate under natural sunlight intensity conditions. These studies demonstrated that the reduction in photosynthetic capacity could begin as early as dawn, preceding the apparent midday depression. At present, however, the early morning reduction in photosynthetic capacity has been reported only for forest trees; thus, it remains unclear whether such a pattern also applies to herbs that grow in open habitats of wet temperate regions. Hence, we here report the results of a field experiment in which we investigated the effect of changing light intensity and diurnal changes in photosynthetic light capacity on midday depression in the temperate perennial herb *Oenothera biennis*.

## Results

**Figure 1:**
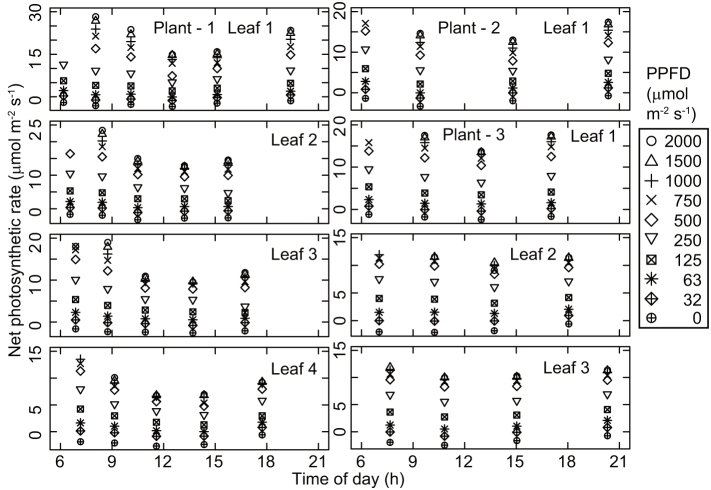
Diurnal course of light response curves of net photosynthetic rate of the leaves. The same set of symbols is used for all panels to indicate the incident LED light intensity on each leaf.

**Figure 2:**
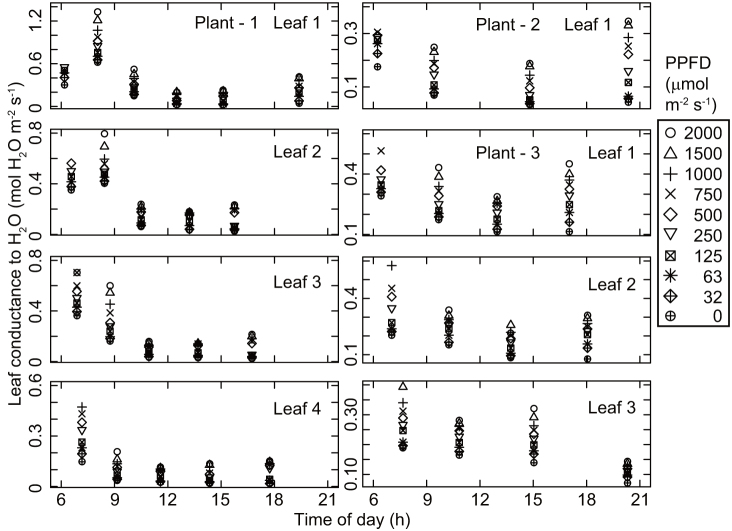
Diurnal course of leaf conductance to H_2_O.

**Figure 3:**
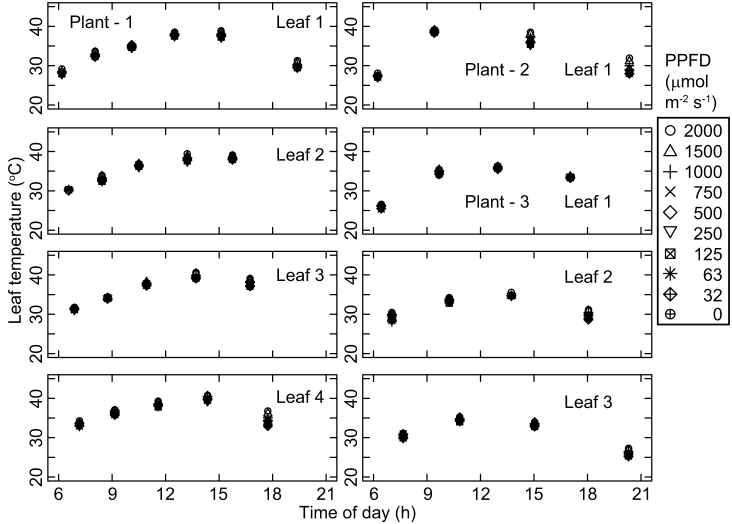
Diurnal course of leaf temperature.

**Figure 4:**
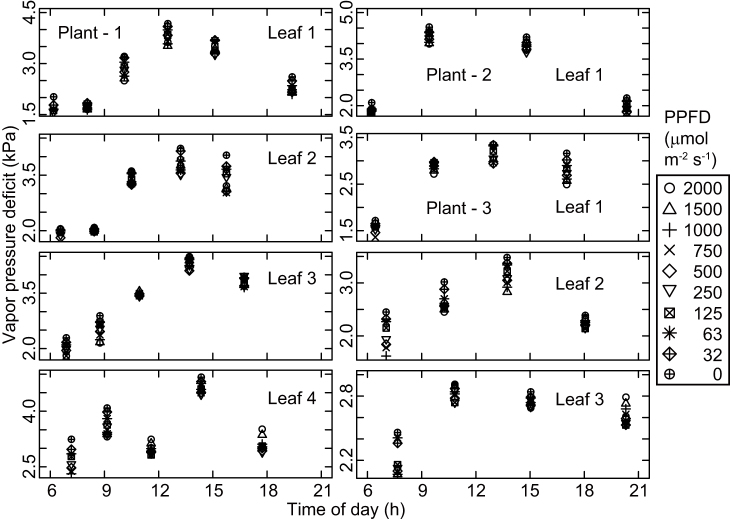
Diurnal course of vapor pressure deficit based on leaf temperature.

**Figure 5:**
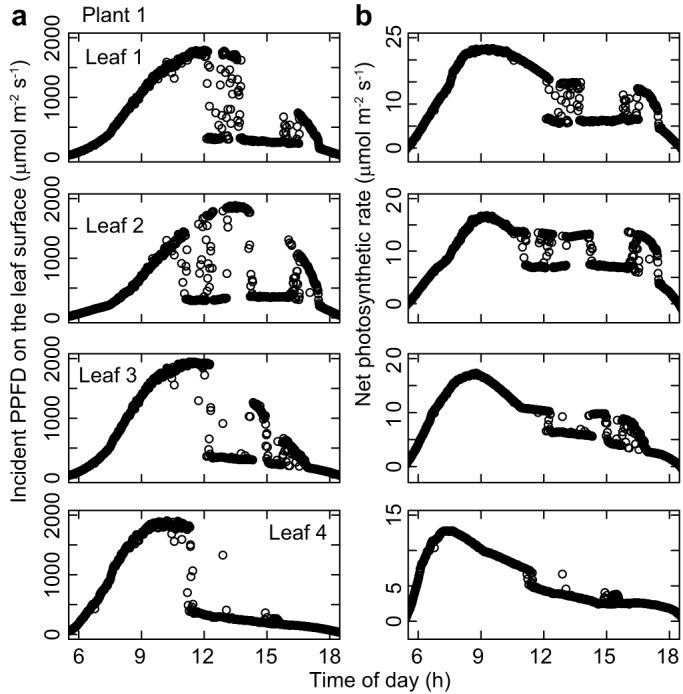
Diurnal course of (a) incident photosynthetic photon flux density (PPFD) on the leaves, and (b) net photosynthetic rate of the leaves for Plant 1.

The observed patterns of diurnal change in the photosynthetic light response curves were similar among the three investigated plants. The photosynthetic rate at each photosynthetic photon flux density (PPFD) value declined from the first measurement taken just after sunrise toward midday (Fig. 1). Photosynthetic rates and stomatal conductance values were lowest around early afternoon (Fig. 1 and 2), when the leaf temperature and vapor pressure deficit were high (Fig. 3 and 4), and then recovered toward sunset. In contrast, incident PPFD increased after sunrise and reached a maximum around noon (Fig. 5a). Combining these two opposing effects, the *in situ* net photosynthetic rate of the leaves increased after sunrise, reached a maximum around mid-morning, and then showed midday depression (Fig. 5b). In the afternoon, the experimental leaves, which faced the southeast direction, were shaded by other leaves on the same stem.

**Figure 6:**
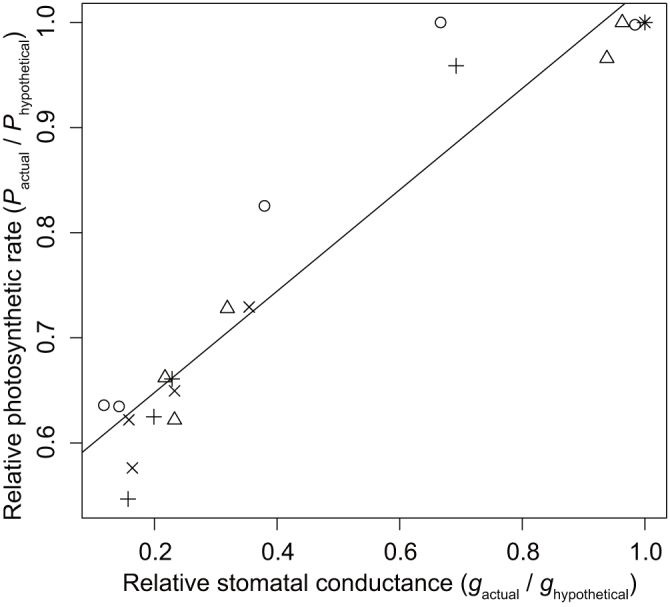
Dependence of relative photosynthetic rate on relative stomatal conductance. The different symbols (circles, crosses, triangles, and pluses) indicate the four different leaves. The solid line indicates significant ordinary least-squares linear regression: *P*_actual_/*P*_hypothetical_ = 0.482 *g*_actual_/*g*_hypothetical_ + 0.551 (P < 1.0 × 10^−10^, *R*^2^ = 0.916, n = 20).

The relative photosynthetic rate was positively correlated with relative stomatal conductance during the day (Fig. 6), indicating that at least part of the reduction in photosynthetic rate during the day was related to stomatal closure. The observed daily net photosynthesis rate was 34% lower on average (range 30–38% for the four leaves) compared with that under the hypothetical situation of no midday depression. In turn, the midday depression reduced daily transpiration by 45% (40–52%) compared with the hypothetical situation. Consequently, the midday depression increased daily water-use efficiency by 22% (9–46%) compared with the hypothetical situation without midday depression.

## Discussion

The reduction in photosynthetic capacity started from as early as sunrise, preceding the apparent midday depression. This reduction was offset by an increase in incident PPFD in the early morning, which explains why the midday depression effect was apparent only at midday. Our result is consistent with reports for two tropical canopy tree species, *Dipterocarpus sublamellatus* and *Neobalanocarpus heimii*^21^,^22^, and for one temperate tree species, *Quercus crispula*^23^. This agreement among experimental results strongly suggests that the latent morning reduction in photosynthetic capacity is a common phenomenon across species. Recent studies have incorporated leaf-level stomatal regulation into process-based models of ecosystem gas exchanges of forest trees^3^,^4^,^5^. Our present results indicated the necessity of incorporating stomatal regulation into models for herbaceous communities as well. Our results, together with the previous results on trees^21^,^22^,^23^, indicate the potential of underestimating photosynthetic capacity even in the morning if the leaves show midday depression at the subsequent midday hours. As the process-based models rely on field measurements of a limited sample of leaves^24^,^25^,^26^, future studies are needed to further investigate the possibility and magnitude of morning reduction for other species in different environments.

Throughout the day, the relative photosynthetic rate was strongly dependent on relative stomatal conductance (Fig. 6). We further showed that stomatal closure increased the water-use efficiency of the leaves, supporting the models of optimal stomatal regulation^15^,^27^,^28^,^29^,^30^,^31^,^32^. In general, midday depression is caused by stomatal limitations and other non-stomatal limitations such as photoinhibition^33^,^34^,^35^,^36^,^37^, photorespiration^33^,^35^, and reduced Rubisco activation under high temperature^38^,^39^. We showed that at least some part of the reduction in photosynthesis was caused by stomatal closure; however, this finding does not preclude the existence of alternative mechanisms that may also have affected photosynthesis.

There are several limitations to the present study. The study was conducted during the hottest season of a single year, and examined only one species in an open site. However, the degree of photosynthetic limitation varies between habitats^35^,^40^,^41^ and differs according to soil water conditions^22^,^37^,^40^,^42^, which was not measured in the present study. In addition, we used LED light sources, and as such, the effects of heat loading^29^,^43^,^44^ may not have been properly evaluated. Further study is needed to clarify to what extent the present results are applicable to other plant species and different environments.

## Methods

### Study site and materials

Our study site consisted of an artificially constructed open-sky empty lot on the campus of Ishikawa Prefectural University, Nonoichi, Ishikawa Prefecture, Japan (36°30′N, 136°35′E). The mean annual temperature and precipitation (2002–2008) at the study site were 14.3°C and 2161 mm, respectively (data from IPU-1 at Ishikawa Prefectural University). The study was conducted in August 2006, during the hottest part of summer. The mean air temperature at the site in August 2006 was 27.6°C. The study species, *Oenothera biennis* L. (Onagraceae), is a monocarpic perennial herb that was introduced to Japan from North America, and it is a pioneer species in open, disturbed habitats, such as roadsides, in Japan^45^. Three single-stem individual bolting rosettes (i.e., vertical stems, 0.8–1.3 m tall) that were naturally growing at the site were measured *in situ*. These three plants, hereafter referred to as Plants 1, 2, and 3, were isolated and had no shading from neighbors.

### Measurement of photosynthesis

We measured the photosynthetic rates of a total of eight leaves from the three plants on August 3 (Plant 1), August 24 (Plant 2), and September 4 (Plant 3), 2006 using a portable photosynthesis system (LI-6400; LI-COR, Lincoln, USA) equipped with an LED light source (LI-6400-02B). For each leaf, photosynthetic light response curves were repeatedly measured (4–6 times) from dawn to dusk. During all but the first (dawn) measurement for each leaf, the incident PPFD on the leaf surface was lowered progressively (2000, 1500, 1000, 750, 500, 250, 125, 63, 32, and 0 μmol m^−2^ s^−1^). Incident PPFD, supplied as natural sunlight to each leaf surface, was determined prior to the dawn measurements using a quantum sensor (IKS-27; KOITO Kogyo, Yokohama, Japan) temporarily placed on the center of each leaf lamina. During the dawn measurements, the highest PPFD supplied by the LED was controlled so that it did not exceed the recorded *in situ* incident PPFD to avoid artificially stimulating the leaves with excessively high light intensities. During each measurement, the PPFD was kept constant at each decreasing level until equilibration. Leaf conductance to H_2_O (*g*_actual_; mol m^−2^ s^−1^), leaf transpiration rate (*Tr*_actual_; mmol H_2_O m^−2^ s^−1^), leaf temperature, and vapor pressure deficit based on leaf temperature were simultaneously calculated using the LI-6400 system. In the following analyses, leaf conductance was regarded as equivalent to leaf stomatal conductance, assuming that the leaf boundary layer resistance was negligible. The CO_2_ concentration of the air entering the leaf chamber was controlled at 350 ppm.

### Measurement of incident PPFD

**Figure 7:**
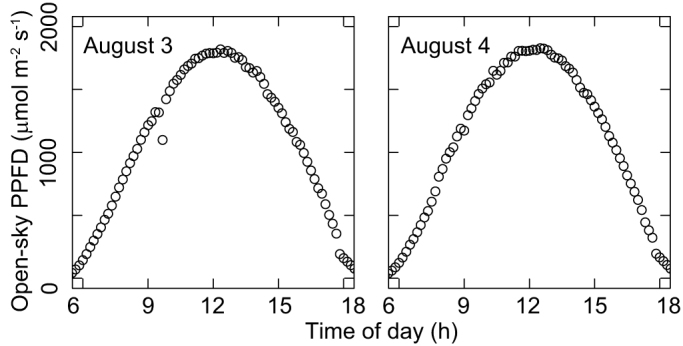
Diurnal changes in open-sky PPFD on the two measurement days in 2006.

We chose two consecutive clear days with very similar weather to conduct measurements (Fig. 7). For Plant 1, a diurnal course of photosynthetic light response curves was measured on the first day (August 3) as described above, and a diurnal course of light intensity on the same leaves was measured on the second day (August 4). Light data from August 4 were used as an estimate for photosynthesis on August 3, assuming that the weather conditions were the same over these two days. For Plants 2 and 3, rainy or cloudy weather prevailed after the photosynthetic measurements, so neither incident light intensity nor the photosynthetic rate under natural sunlight was estimated. We used small, leaf-mounted gallium arsenide photodiodes (150 mg: G1118; Hamamatsu Photonics, Hamamatsu, Japan) to estimate incident PPFD on the leaves as described by Nishimura, et al.^46^. Photodiodes were calibrated individually against a quantum sensor (IKS-27; Koito, Yokohama, Japan). After measurement of photosynthetic light responses, one photodiode was mounted on the adaxial surface of each leaf by using adherent tape. The photodiodes were connected to a voltage logger (Thermodac-F; Eto Denki, Tokyo, Japan) using light telephone wires. The incident PPFD on each leaf was recorded every 10 min for 24 h.

### Data analysis

As described above, the photosynthetic rate under high light intensity was not quantified during the first (dawn) measurement for each leaf. To obtain these data, we fitted a non-rectangular hyperbola^47^ to each of the PPFD-photosynthesis relationship models to estimate the photosynthetic rate at high PPFD ranges (*R*^2^ > 0.999 for all cases). We also fitted an empirical hyperbola^9^ to each of the PPFD-*g*_actual_ relationship models (*R*^2^ > 0.965 for all cases) and the PPFD-*Tr*_actual_ relationship models (*R*^2^ > 0.981 for all cases) to estimate *g*_actual_ and *Tr*_actual_ at high PPFD ranges, respectively. Curve-fitting was conducted with R software, version 3.0.0 (R Foundation for Statistical Computing, Vienna, Austria). Next, the photosynthetic rate, stomatal conductance, and transpiration rate were estimated by linear interpolation for any PPFD intensity and values between the two successive measurements for each leaf. Pre-dawn and post-sunset photosynthetic response curves were assumed to be the same as those obtained for the first and final measurements, respectively. The net photosynthetic rate for every 10-min increment was calculated for each leaf from the estimated light response curves and the estimated incident PPFD. The dark respiration rate was estimated as the absolute value of the net photosynthetic rate at PPFD = 0. The gross photosynthetic rate at each moment (*P*_gross_actual_) was calculated as the sum of the net photosynthetic rate and the dark respiration rate at that moment. Instantaneous transpiration rates and stomatal conductance at each moment were calculated for each leaf from the incident PPFD data in the same manner as described for the photosynthetic rate. The sum of daily net photosynthesis (*P*_day_actual_) and transpiration (*Tr*_day_actual_) was calculated by integrating these values.

We also simulated the hypothetical situation without diurnal changes in photosynthetic and stomatal light response curves^48^. For this scenario, the gross photosynthetic rate and stomatal conductance for each light intensity value were kept constant at their daily maximum values, which were observed in the early morning (see Results). Diurnal changes in dark respiration were maintained at their actual values. The instantaneous net (*P*_hypothetical_) and gross (*P*_gross_hypothetical_) photosynthetic rates, stomatal conductance (*g*_hypothetical_), and transpiration rate (*Tr*_hypothetical_) at each moment were then calculated as described above, using the same set of incident PPFD data. These results were integrated over the day to calculate daily photosynthetic and respiration rates under the hypothetical situation (*P*_day_hypothetical_ and *Tr*_day_hypothetical_). The daily water-use efficiency of each leaf was calculated as the daily net photosynthetic rate divided by the daily transpiration rate for the actual and hypothetical scenarios. Finally, we calculated the instantaneous relative gross photosynthetic rate (*P*_gross_actual_/*P*_gross_hypothetical_)^42^ and relative stomatal conductance (*g*_actual_/*g*_hypothetical_)^42^ as the ratios between the actual and hypothetical values, respectively. These relative values were calculated using the incident natural PPFD intensity for each moment when the photosynthetic light response curve was directly measured. The dependence of *P*_gross_actual_/*P*_gross_hypothetical_ on *g*_actual_/*g*_hypothetical_ was tested with ordinary least-squares regression using the software R.

## Author Contributions

K.K. performed the experiments and analyzed the data; K.K. and S.T. wrote the paper.
